# FGL1 and FGL2: emerging regulators of liver health and disease

**DOI:** 10.1186/s40364-024-00601-0

**Published:** 2024-05-31

**Authors:** Jiongming Chen, Lei Wu, Yongsheng Li

**Affiliations:** 1 Chongqing University Cancer Hospital, School of Medicine, Chongqing University, Chongqing, 400030 China; 2 Department of Medical Oncology, Chongqing University Cancer Hospital, Chongqing, 400030 China

**Keywords:** FGL1, FGL2, Liver disease, Therapeutic target, Immunotherapy, Biomarker

## Abstract

Liver disease is a complex group of diseases with high morbidity and mortality rates, emerging as a major global health concern. Recent studies have highlighted the involvement of fibrinogen-like proteins, specifically fibrinogen-like protein 1 (FGL1) and fibrinogen-like protein 2 (FGL2), in the regulation of various liver diseases. FGL1 plays a crucial role in promoting hepatocyte growth, regulating lipid metabolism, and influencing the tumor microenvironment (TME), contributing significantly to liver repair, non-alcoholic fatty liver disease (NAFLD), and liver cancer. On the other hand, FGL2 is a multifunctional protein known for its role in modulating prothrombin activity and inducing immune tolerance, impacting viral hepatitis, liver fibrosis, hepatocellular carcinoma (HCC), and liver transplantation. Understanding the functions and mechanisms of fibrinogen-like proteins is essential for the development of effective therapeutic approaches for liver diseases. Additionally, FGL1 has demonstrated potential as a disease biomarker in radiation and drug-induced liver injury as well as HCC, while FGL2 shows promise as a biomarker in viral hepatitis and liver transplantation. The expression levels of these molecules offer exciting prospects for disease assessment. This review provides an overview of the structure and roles of FGL1 and FGL2 in different liver conditions, emphasizing the intricate molecular regulatory processes and advancements in targeted therapies. Furthermore, it explores the potential benefits and challenges of targeting FGL1 and FGL2 for liver disease treatment and the prospects of fibrinogen-like proteins as biomarkers for liver disease, offering insights for future research in this field.

## Introduction

Liver disease encompasses a range of liver-related disorders that have increasingly become a significant global health concern, posing a substantial threat to human well-being [1, 2]. According to the World Health Organization (WHO), the worldwide prevalence of hepatitis B virus (HBV) and hepatitis C virus (HCV) stands at 3.5% and 1%, respectively, with approximately 3 million new cases reported annually [3]. In 2022, there were 0.87 million new liver cancer cases globally, making it the sixth most common newly diagnosed cancer [4]. Liver disease claims over 2 million lives yearly, representing about 4% of all global deaths, with cirrhosis and liver cancer ranking among the top 20 causes of death [5, 6]. Liver disease has emerged as a critical public health issue in many developing nations [7], with around one-fifth of China’s population affected [8, 9]. Given the high incidence and mortality rates associated with liver disease, there is an urgent need for the development of new treatments to address this pressing situation.

Fibrinogen-related proteins (FREPs) are a group of proteins characterized by the presence of fibrinogen-related domains (FReDs) [10]. Structurally, they typically contain multiple fibrinogen-like domains that form fibrinogen-like globular domains at the C-terminus with significant homology. These proteins are widely expressed in mammals and serve important physiological roles [11]. Recent research has highlighted the involvement of fibrinogen-like protein 1 (FGL1) and fibrinogen-like protein 2 (FGL2), both members of the FREPs family, in the regulation of various liver diseases [12, 13]. FGL1, also known as hepassocin (HPS) or hepatocyte-derived fibrinogen-related protein 1 (HFREP-1), is a liver-secreted hepatokine that influences proliferation and metabolism [14–16]. Its expression notably increases following partial hepatectomy (PHx), facilitating liver regeneration [17]. FGL1 exhibits a dual role in hepatocellular carcinoma (HCC). Initial studies demonstrated its ability to impede HCC progression [18], while recent findings have identified FGL1 as a functional ligand of lymphocyte activation gene 3 (LAG-3), thereby promoting HCC progression through modulation of the tumor microenvironment (TME) [19]. On the other hand, FGL2, initially recognized for its clotting-related functions, is a versatile protein with various roles [20]. Subsequent studies have revealed its capacity to hinder dendritic cell (DC) maturation and T&B cell proliferation, as well as to stimulate T&B cell apoptosis. Moreover, it inhibits the differentiation of non-antigen-dependent B cells into plasma cells and diminishes plasma cell viability, leading to immunosuppression [21–25]. Deletion of soluble FGL2 (sFGL2) has been shown to alleviate the inhibition exerted by regulatory T cells (Tregs) on effector T cell proliferation and enhance the responsiveness of T cells, B cells, and DCs [26, 27]. This protein plays a crucial immunomodulatory role in viral-induced inflammation, autoimmune diseases, xenograft rejection, and tumors [13, 28, 29], particularly in liver diseases [30–32].

This review provides an overview of the current knowledge regarding the roles of FGL1 and FGL2 in liver disease. Additionally, we discuss the potential of FGL1 and FGL2 as immunotherapeutic targets for various liver diseases.

## Structure and function

### FGL1

**Fig. 1:**
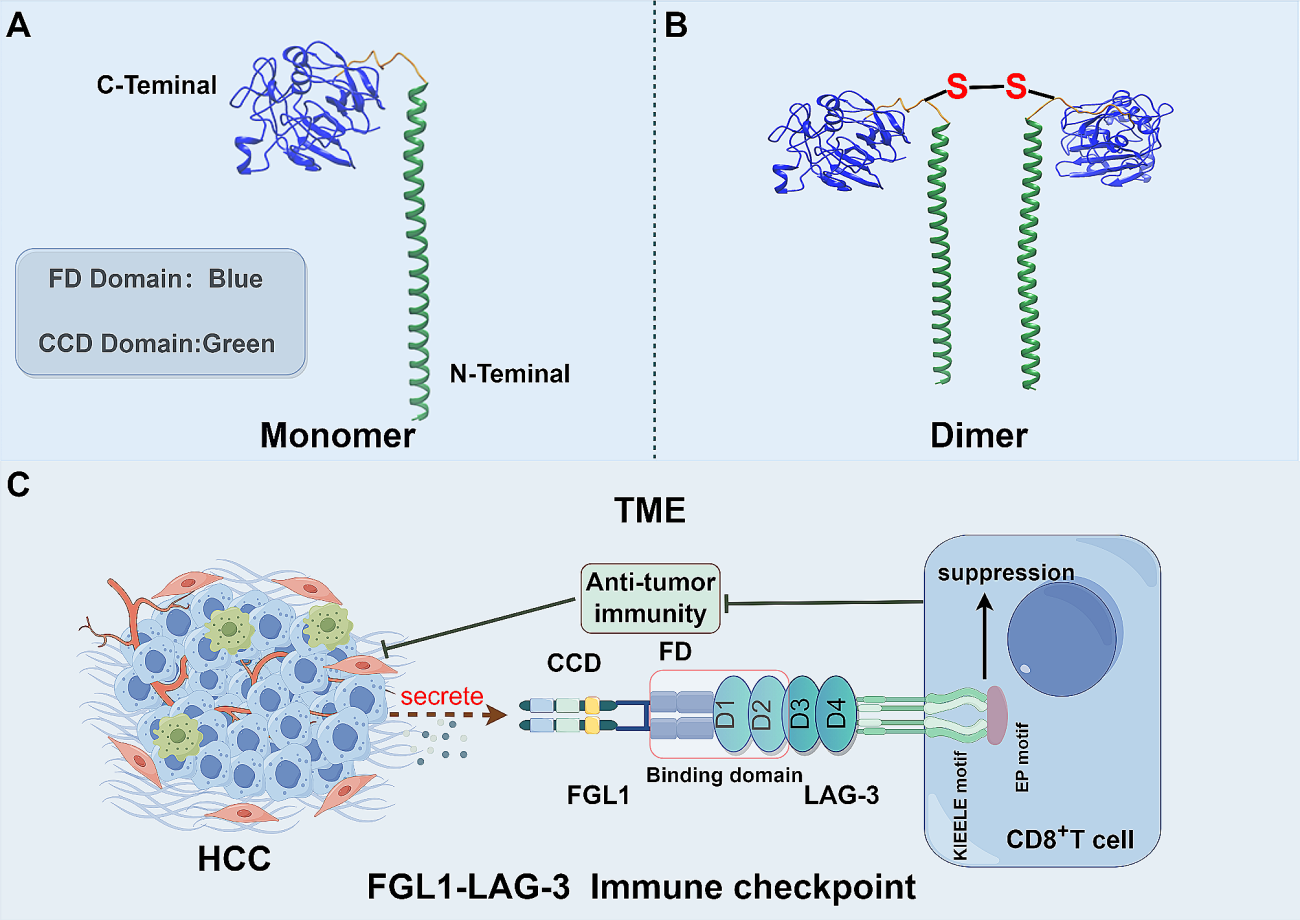
Structure of the FGL1 and FGL1-LAG-3 immune checkpoint. (**A**) The FGL1 monomer is composed of an N-terminal CCD domain and a C-terminal FD domain. (**B**) When two monomers of FGL1 combine, they form a dimer linked by a disulfide bond. (**C**) The FD structural domain of FGL1, along with the D1 and D2 structural domains in LAG-3, serve as the binding sites for LAG3 and FGL1. In HCC, the secretion of FGL1 binds to LAG-3 on the surface of CD8^+^ T cells, leading to the inhibition of T cell activation and the promotion of tumor progression

FGL1 belongs to the fibrinogen superfamily [33]. The human FGL1 gene was mapped to chromosome 8 and initially discovered by Yamamoto et al. using a cDNA library constructed with mRNA from human HCC specimens [34, 35]. It encodes a 312-amino acid protein with a sequence that shares significant homology with the β and γ subunits of fibrinogen and other FREPs. However, it lacks platelet-binding sites, cross-linking regions, and thrombin-sensitive sites related to coagulation [35, 36]. Normally, FGL1 is primarily secreted by liver cells and is minimally expressed in pancreatic tissues [19]. The FGL1 protein consists of a coiled-coil domain (CCD) signaling recognition peptide at the N-terminus and a functional peptide with a fibrinogen-like structural domain (FD) at the C-terminus [15] (Fig. 1A). UniProt data indicates that the amino acid sequence range for CCD is from 23 to 61, while FD’s range spans from 74 to 306. Furthermore, Hara et al. identified a rat homolog of the FGL1 gene that shares approximately 80% homology with the human FGL1 gene, encoding a 314-amino acid protein. This protein forms a homodimer linked by disulfide bonds, with molecular weights of 66 kDa and 34 kDa under non-reducing and reducing conditions, respectively [37] (Fig. 1B).

FGL1 is a secreted protein with mitogenic activity that promotes hepatocyte mitosis through autocrine mechanism, playing a crucial role in hepatocyte regeneration [17, 37, 38]. The role of FGL1 in HCC is debated. Some early studies suggest that FGL1 is down-regulated in human liver cancer tissues [19], inhibiting HCC development through various mechanisms [18, 39–41]. However, more recent research indicates that FGL1 may actually promote HCC progression [42–44]. The FD domain of FGL1 binds to the D1 and D2 immunoglobulin superfamily domains of LAG-3 extracellular region, leading to T cell inhibition and depletion [19, 45, 46] (Fig. 1C). Upstream signaling pathways, such as interleukin (IL)-6/signal transducer and activator of transcription 3 (STAT3) and hepatocyte nuclear factor (HNF) 1, play a role in regulating FGL1 expression [36]. The presence of post-translational protein modifications and the significant heterogeneity of HCC contribute to the ongoing debate surrounding FGL1’s expression and function in HCC [47, 48]. Additionally, FGL1 has been implicated in metabolic diseases, including promoting insulin resistance [49–51] and liver lipid metabolism disorder [52–54].

### FGL2

FGL2 was initially identified in cytotoxic T lymphocytes (CTLs) and shows a high level of similarity across different species [55]. The FGL2 gene is found on chromosomes 7 and 5 in humans and mice, respectively, with the encoded proteins having over 80% homology [56]. The human FGL2 gene consists of two exons [57], with a relatively simple gene structure, and gene expression regulation primarily occurs in the FGL2 promoter. Several transcription factors, including CCAAT/enhancer binding protein (C/EBP), Specificity protein 1 (SP1), E26 transformation-specific (ETS), activator protein-1 (AP1), Ikaros, and T cell factor 1 (TCF1), can bind to the FGL2 promoter via cis element consensus sequences to regulate transcription in various cell types. C/EBPα is responsible for the basal expression of FGL2 in hepatocytes, while C/EBPβ controls FGL2 expression in macrophages [20, 58].

**Fig. 2:**
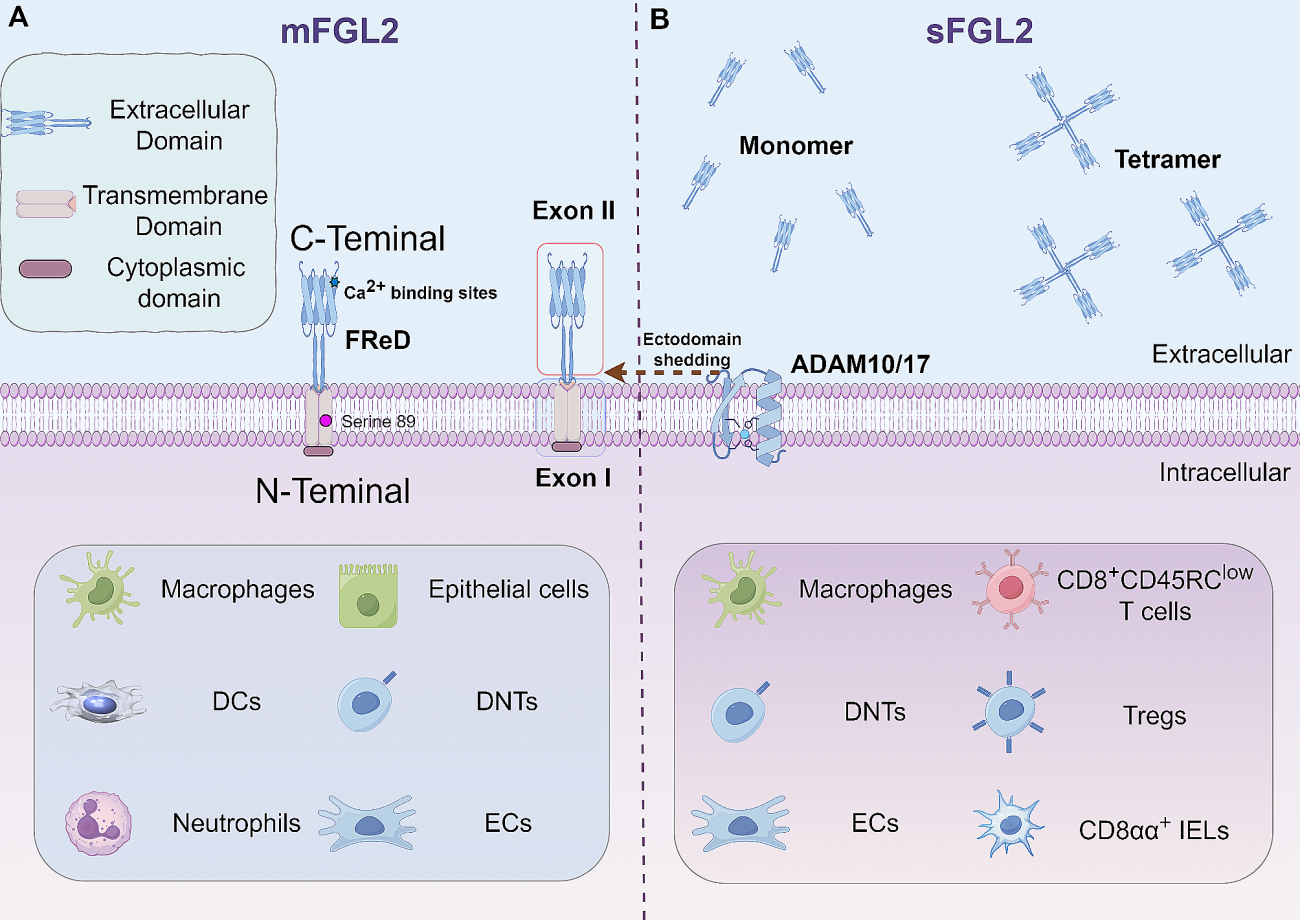
Structure of the FGL2. (**A**) The FGL2 gene consists of two exons. The mFGL2 protein is a type II transmembrane glycoprotein that includes intact intracellular, transmembrane, and extracellular structural domains. The N-terminal linear coiled-coil domain of mFGL2 is responsible for its prothrombinase activity. (**B**) In the presence of the metalloproteinases ADAM10/17, mFGL2 sheds its extracellular domains to generate sFGL2. sFGL2 is present in its native state as both a monomer and a tetramer, with its immunomodulatory function attributed to the C-terminal FReD domain

Koyama et al. provided the initial molecular characterization of FGL2, revealing a 36% similarity to the C-terminus sequence of the β and γ subunits of fibrinogen [55]. The human FGL2 gene encodes a 439-amino acid protein, with the first 204 amino acids being encoded by exon I [28]. The N-terminus consists of a cytoplasmic domain of 2 amino acids and a 21-amino acid transmembrane domain, acting as a signal peptide. Within the N-terminus domain, two α-helical chains intertwine to create a mechanical superhelical cable structure [20] (Fig. 2A). Cysteine plays a crucial role in forming tertiary or quaternary structures through interchain disulfide bonds in this domain [59, 60]. The conserved sequence of 229 amino acids in the C-terminus forms a globular domain known as FReD. FReD plays a key role in determining the functional specificity and complexity of FGL2, including receptor recognition, calcium binding, substrate binding, and immune regulation [61]. Four cysteines (206, 235, 364, and 377) within FReD maintain the functional structure of FGL2, forming an essential immunoregulatory domain [21, 62, 63]. The three amino acids (362, 364, and 366) near the C-terminus constitute the Ca^2+^ binding site. Adjacent to the Ca^2+^ binding site, five amino acids (371, 374, 375, 384, and 385) form the polymerization pocket of each monomer and contribute to the polymerization of FReD [60].

FGL2 is primarily found in lymphoid tissues like the spleen and lymph nodes, with its consistent presence during both embryonic development and in mature tissues suggesting a crucial physiological role [29]. However, excessive FGL2 expression in response to tumors and infections can lead to complement activation, inflammatory responses, and immune dysfunction. Conversely, a complete lack of FGL2 can also be detrimental, as it may result in autoimmune diseases and acute rejection of xenografts [13, 61, 64], underscoring the significance of FGL2 in the body. There are two forms of FGL2 proteins - transmembrane FGL2 (mFGL2) and sFGL2 - each with distinct structures and functions [65]. Our research team observed that during the resolution of inflammation, A Disintegrin And Metalloproteinase (ADAM) 10 and 17 facilitate the shedding of the outer domain of FGL2, leading to the secretion of sFGL2 [66, 67]. This sheds light on the process of transformation from mFLG2 to sFGL2.

### mFGL2

mFGL2 is a type II transmembrane glycoprotein that is expressed on the surface of various cells, such as macrophages, epithelial cells, endothelial cells (ECs), DCs, neutrophils, and CD3^+^CD4^−^CD8^−^T cells (DNTs) [28, 68–70]. It consists of both the N-terminal transmembrane region and the C-terminal extracellular domain. The N-terminal structure of mFGL2 is implicated in immune-related coagulation, with serine residues at position 89 in this domain playing a role in its pro-coagulation activity [29, 71]. The mFGL2 exhibits serine protease activity and is capable of directly cleaving prothrombin into thrombin independently of coagulation factors VII or X [20, 72]. Functionally, FGL2 cleaves prothrombin to thrombin via a non-classical pathway, after which thrombin interacts with phospholipids, free calcium, and coagulation factor Va on the cell membrane to initiate the coagulation cascade, resulting in local fibrin deposition, intravascular thrombosis, and tissue inflammation. This process can be inhibited by the specific serine protease inhibitor diisopropylfluorophosphate [20, 60, 62, 71].

The prothrombin activity of mFGL2 plays a crucial role in the pathogenesis of various conditions such as pathogen infection, tumors, abortion, and xenograft rejection [66, 72–77]. Specifically, it can induce fiber deposition in hepatitis and liver fibrosis [72, 78], as well as angiogenesis in HCC [65], potentially worsening disease progression. Studies have shown that tumor necrosis factor-alpha (TNF-α) triggers the activation of nuclear factor-kappaB (NF-κB) and p38 mitogen-activated protein kinase (MAPK) signaling pathways in Cerebral Microvascular Endothelial Cells (CMECs), leading to increased mFGL2 expression, which in turn causes microvascular dysfunction and microthrombosis [79]. Furthermore, research has demonstrated that FGL2 promotes the proliferation and angiogenesis of HCC by stimulating thrombin production, subsequently activating protease-activated receptors (PARs) and downstream signaling pathways [80]. mFGL2 not only enhances the classical inflammatory cascade but also serves as a co-receptor to facilitate signal transduction with Toll-like Receptors (TLRs) or other Pattern Recognition Receptors (PRRs) [81]. Additionally, some studies have found that mFGL2 can activate Toll-like receptor 4 (TLR4) and downstream pathways in liver macrophages to regulate hepatic lipid metabolism [82].

### sFGL2

sFGL2, a protein mainly secreted by various immune cells such as Tregs, macrophages, and ECs [21, 22] (Fig. 2B), was first discovered in the supernatant of peripheral blood mononuclear cells (PBMCs) culture medium [83]. Recent studies have shown its expression in specific subpopulations of Tregs in mice, including CD8^+^CD45RC^low^T cells [84], CD8αα^+^ intraepithelial lymphocytes (IELs) [85], and DNTs [26], with the protein existing in both monomer and tetramer forms. Oligomerization of FGL2 is promoted by specific cysteines at positions 94, 97, 184, and 187, while glycosylation of certain amino acids at sites 172, 228, 256, and 329 maintains the solubility of sFGL2. Oligomers have lower biological and immunosuppressive activity compared to monomers but show a stronger affinity with antigen-presenting cells (APCs) [63]. In contrast to mFGL2, which exerts procoagulant activity through its N-terminal domain, sFGL2 primarily uses the C-terminal FReD to regulate immunity [86]. Peptide blockade assays have confirmed that the active sites of sFGL2 are predominantly located in the FReD [63].

Fc gamma receptor IIB (FcγRIIB) and Fc gamma receptor III (FcγRIII) are specific receptors for sFGL2 and play a crucial role in the FGL2 regulatory pathway [87]. FcγRIIB, uniquely, contains an immunoreceptor tyrosine-based inhibition motif (ITIM) in its intracellular domain [88], making it the sole Fc gamma receptor (FcγR) with inhibitory function [89]. On the other hand, FcγRIII possesses an immunoreceptor tyrosine-based activation motif (ITAM) that facilitates activation signal transduction [90]. Following sFGL2 binding to FcγRs, it modulates the expression ratio or affinity of FcγRIIB and FcγRIII on the cell surface, thereby exerting specific regulatory effects [91].

## Research progress of fibrinogen-like proteins in liver disease

Recent studies have highlighted the critical regulatory roles of Fibrinogen-like proteins in various liver diseases. FGL1 is primarily associated with liver regeneration, non-alcoholic fatty liver disease (NAFLD), and liver cancer, while FGL2 is known to be involved in viral hepatitis, liver fibrosis, HCC, and liver transplantation.

### The role of FGL1 in liver diseases

#### FGL1 in liver injury and repair

**Fig. 3:**
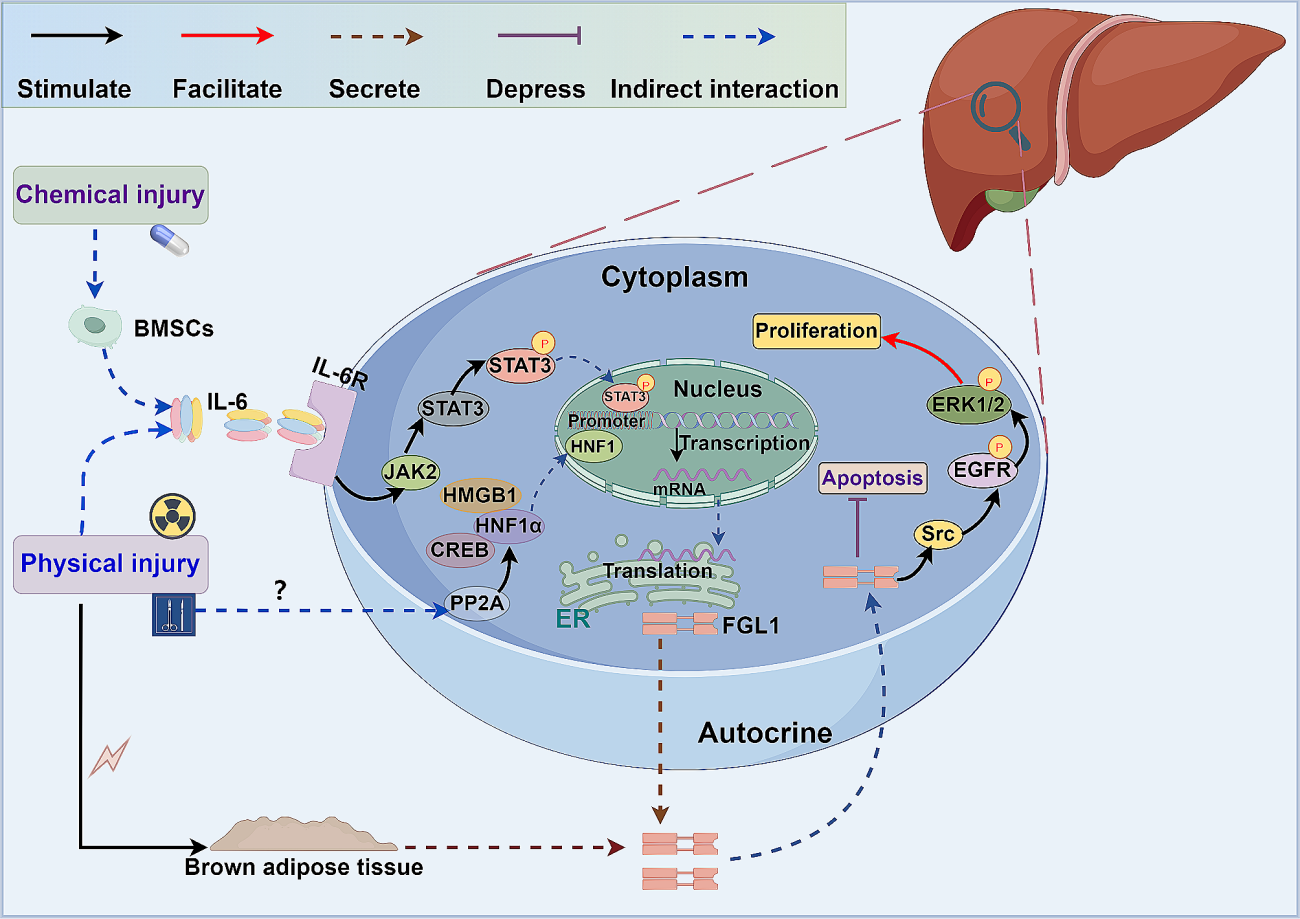
The roles of FGL1 in liver injury and repair. Physical and chemical injuries induce activation of the JAK2-STAT3 pathway via the inflammatory factor IL-6. Furthermore, physical injuries facilitate the formation of a complex between HNF1α, HMGB1, and CREB in the cytoplasm through PP2A. Subsequently, the pSTAT3 and HNF1α/HMGB1/CREB transcriptional complex translocates into the nucleus to enhance the transcription of FGL1. FGL1, in turn, triggers the Src/EGFR/ERK1/2 pathway via an autocrine mechanism, thereby regulating hepatocyte proliferation and apoptosis. Moreover, FGL1 expression in brown adipose tissue is upregulated following physical injury, contributing to the repair process of liver injury

Hepatocytes, typically dormant cells, possess a remarkable capacity for regeneration [92]. Following physical or chemical damage to the liver, a variety of cytokines and growth factors are released either by the liver itself or other organs to kickstart and regulate the process of liver regeneration [93]. FGL1, a crucial hepatokine secreted by hepatocytes, stimulates hepatocyte division through autocrine mechanisms post-liver injury, thereby overseeing liver regeneration [17] (Fig. 3). Research by Yu et al. demonstrated that following PHx, the Janus kinase 2 (JAK2)/STAT3 signaling pathway can be activated via the IL-6/IL-6 Receptor (IL-6R) pathway, leading to increased FGL1 promoter activity. Moreover, this activation can trigger the HNF1α - high mobility group box 1 (HMGB1) - cAMP response element-binding protein (CREB) transcription complex to further modulate FGL1 transcription [94]. Another study focusing on hyperglycemic crisis revealed that elevated blood glucose levels could boost STAT3 and Protein Phosphatase 2 A (PP2A) - HNF1 pathway activity, subsequently inducing FGL1 expression and mitigating the detrimental effects of high blood glucose on hepatocytes [50]. Furthermore, researchers noted a significant upregulation of FGL1 in cases of radiation-induced liver damage (RILD), suggesting its involvement in the repair process of RILD. Plasma levels of FGL1 could potentially serve as a biomarker for assessing RILD [95].

Various studies have demonstrated the protective role of FGL1 in drug-induced liver injury (DILI). Yun et al. observed higher expression of FGL1 in the drug-resistant group compared to the drug-sensitive group in a liver injury model induced by carbon tetrachloride (CCL4), suggesting its involvement in the regulation of DILI. FGL1 has potential as a biomarker for predicting liver injury and identifying individuals susceptible to drug-induced hepatotoxicity [96]. In another study on CCL4-induced liver injury, it was found that mesenchymal stem cell (MSC) conditioned medium could induce FGL1 expression through the IL-6 pathway, reducing early hepatocyte apoptosis and liver injury [97]. Further investigations revealed that bone marrow mesenchymal stem cells (BMSCs) promoted FGL1 expression by activating the STAT3 signaling pathway in rat models of acute liver injury induced by D-galactosamine (D-Gal) and lipopolysaccharide (LPS). FGL1 was shown to inhibit the upregulation of pro-apoptotic factors and enhance the expression of anti-apoptotic factors, ultimately reducing hepatocyte necrosis, apoptosis, and inflammatory cell infiltration, while promoting liver regeneration and improving liver function [98, 99]. Additionally, Cao et al. identified FGL1 receptors on the liver cell membrane [18], with FGL1 inducing extracellular signal-regulated kinases (ERK) 1/2 phosphorylation via the autocrine pathway to stimulate hepatocyte proliferation [99]. Subsequent research further elucidated the molecular mechanism through which FGL1 triggers activation of the ERK pathway. In the L02 cell line, FGL1 stimulates phosphorylation of the epidermal growth factor receptor (EGFR), leading to the activation of the non-receptor tyrosine kinase Src. When EGFR expression was down-regulated using gefitinib or EGFR-specific siRNA, the activation of the ERK pathway induced by FGL1 was inhibited. Moreover, inhibiting Src effectively blocked the activation of EGFR by FGL1, highlighting the crucial role of the FGL1/Src/EGFR/ERK signaling pathway in the regulation of hepatocyte proliferation [100].

#### FGL1 in NAFLD

NAFLD is defined by the abnormal accumulation of lipid in the liver without the presence of alcohol abuse [101]. Nonalcoholic steatohepatitis (NASH), a type of NAFLD characterized by inflammation [102], is marked by hepatic steatosis, inflammation, hepatocellular injury, and varying degrees of fibrosis [103]. NAFLD is seen as a spectrum of liver abnormalities ranging from nonalcoholic fatty liver (NAFL) to NASH [104]. Due to lifestyle changes in recent years, NAFLD has emerged as the most prevalent chronic liver disease, accounting for approximately 25% of adult liver diseases [105].

**Fig. 4:**
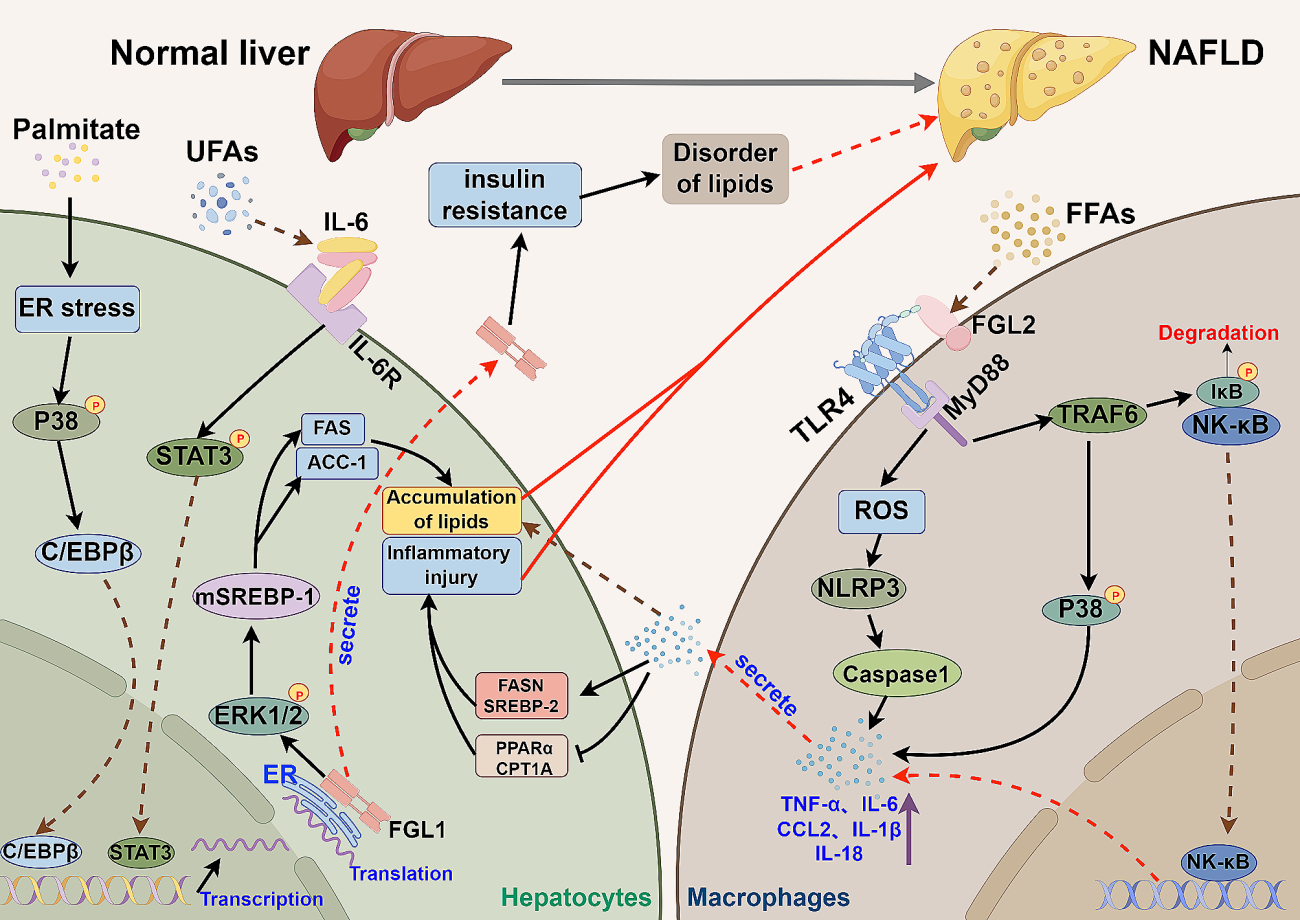
The roles of FGL1 and FGL2 in NAFLD. Palmitate acid and UFA promote FGL1 expression in hepatocytes through the ER stress/P38/C/EBPβ and IL-6/STAT3 pathways, respectively. FGL1 induces lipid accumulation and inflammatory damage via ERK1/2-dependent signaling pathways. FFA induces the expression of FGL2 in the liver, which binds to TLR4 on macrophages and activates pro-inflammatory pathways with MyD88, leading to the production of inflammatory factors like TNF-α, IL-6, CCL2, IL-1β. These factors further contribute to hepatic lipid accumulation and inflammatory injury, ultimately playing a role in the development of NASH. Additionally, FGL1 secreted by hepatocytes can also impact NASH pathology through glycolipid metabolism

Glycolipid metabolism disorder is identified as a prominent factor in the pathogenesis of NAFLD [106]. Recent research has highlighted the significant role of FGL1 in metabolic disorders, particularly in the regulation of blood lipids and glucose metabolism. Wu et al. demonstrated that FGL1 disrupts insulin signaling and induces insulin resistance through modulation of the ERK1/2 pathway [49]. Moreover, Palmitic acid has been shown to upregulate FGL1 expression in primary hepatocytes via C/EBP-β mediated transcriptional activation, leading to the phosphorylation of C-Jun N-terminal kinases (JNKs) and subsequent insulin resistance in skeletal muscle cells [51]. Studies have also reported elevated levels of FGL1 in obese individuals [107], suggesting its potential as a biomarker for metabolic disorders. Additionally, Abdelmoemen et al. observed a significant increase in FGL1 levels in NAFLD patients with type 2 diabetes, attributing to accelerated disease progression through enhanced lipid accumulation in the liver [53]. Furthermore, Wu et al. found that serum FGL1 levels were notably higher in NAFLD patients compared to non-NAFLD individuals. In a high-fat diet (HFD)-induced NAFLD model, overexpression of FGL1 exacerbated liver lipid accumulation and NAFLD activity score (NAS). Mechanistically, FGL1 was found to enhance ERK1/2 phosphorylation, leading to increased expression of mature sterol regulatory element-binding protein 1 (mSREBP-1) and lipid synthesis-related enzymes like fatty acid synthase (FAS) and acetyl-CoA carboxylase-1 (ACC-1), thereby promoting hepatic lipid accumulation and driving NAFLD progression [52]. Moreover, unsaturated fatty acids (UFAs) have been shown to activate the IL-6/STAT3 pathway, resulting in upregulation of FGL1 expression, further contributing to NAFLD development [54] (Fig. 4).

#### FGL1 in liver cancer

Primary liver cancer is the third leading cause of cancer-related deaths. It contributes to 7.8% of total cancer-related deaths worldwide [4, 108]. There are expected to be 1.4 million new cases and 1.3 million deaths by 2040 [109]. The incidence of HCC varies significantly by region, with China representing nearly half of all cases, imposing a substantial medical burden [110]. Currently, early-stage HCC is primarily managed with surgery, while advanced stages often necessitate a combination of radiotherapy, chemotherapy, targeted therapy, and local therapy. As research in immunotherapy for HCC advances, it is emerging as an innovative treatment option [111, 112]. However, the overall response to immunotherapy remains low, posing challenges in reshaping the TME and preventing immune escape in HCC immunotherapy research [113, 114].

**Fig. 5:**
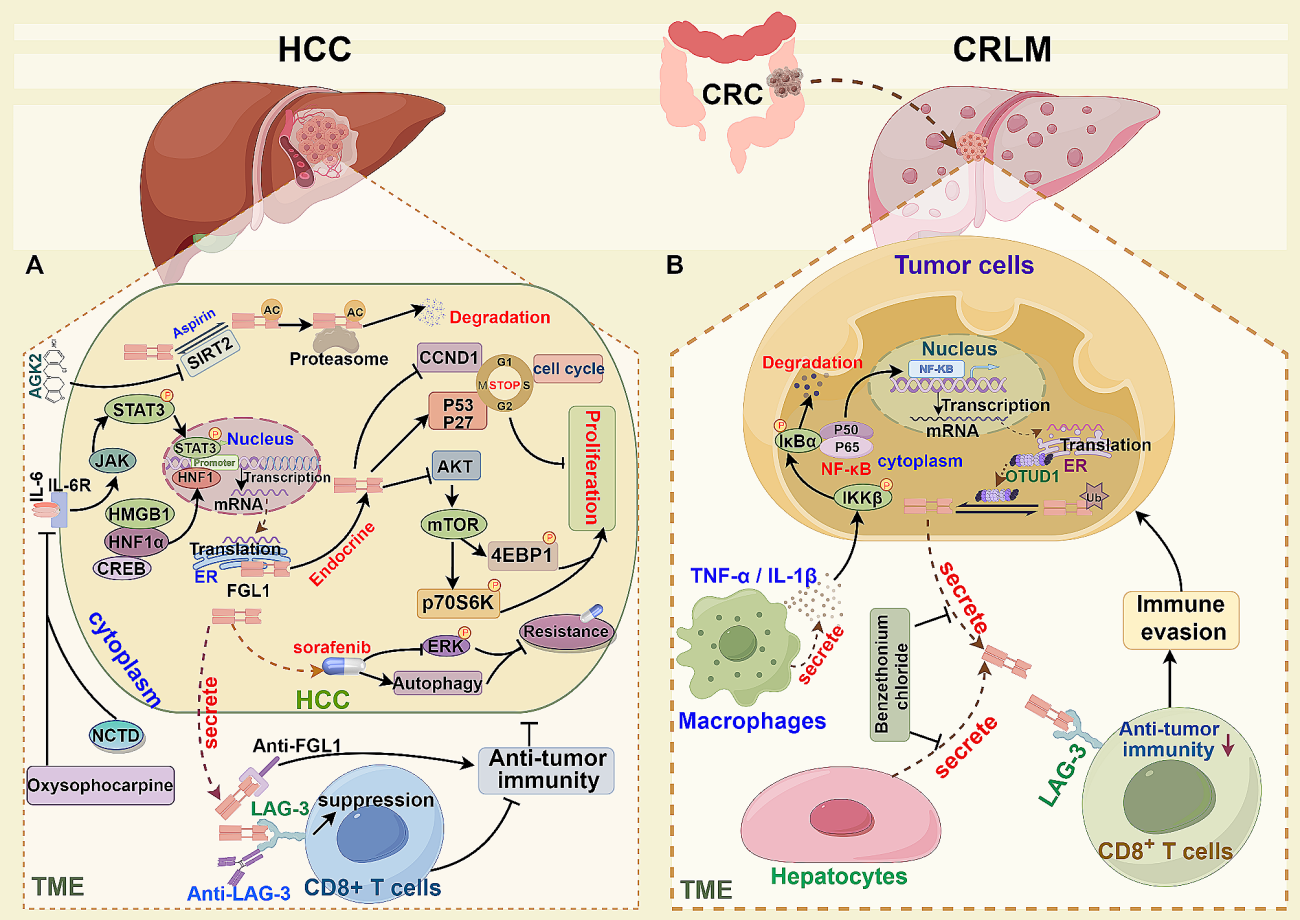
The roles of FGL1 in liver cancer. (**A**) In HCC, the activation of the IL-6/JAK/STAT3 pathway and the HNF1α/HMGB1/CREB transcriptional complex leads to the transcription of FGL1. FGL1 plays a role in inhibiting HCC proliferation by modulating the cell cycle and the AKT/mTOR pathway. Moreover, FGL1 is involved in regulating sorafenib resistance through the ERK and autophagy pathways. HCC-derived FGL1 binds to LAG-3 in CD8^+^ T cells, thereby inhibiting T cell anti-tumor activation. Oxysophocarpine and NCTD work by inhibiting the IL-6/JAK2/STAT3 pathway, suppressing FGL1 expression, and enhancing the immunotherapeutic efficacy of anti-LAG-3. AGK2 and SIRT2 are involved in the regulation of FGL1 degradation by modulating FGL1 acetylation. (**B**) In CRLM, TNFα/IL-1β secreted by TAMs activates the NF-ĸB signaling pathway in tumor cells, up-regulates OTUD1 expression, enhances FGL1 stability through deubiquitination, and further promotes tumor progression by influencing tumor immune escape mechanisms. Benzethonium chloride inhibits tumor growth by targeting the secretion of FGL1

Early studies have indicated that FGL1 may have an inhibitory effect on HCC (Fig. 5A). Chan et al. observed a significant relationship between frequent loss of FGL1 alleles and the development of HCC [34]. Hua et al. noted reduced FGL1 expression in various liver cancer cell lines and clinical samples, with tumor tissue showing lower FGL1 expression compared to para-cancer and normal liver tissue. They also found that FGL1 expression was inversely related to Edmondson grade and metastasis, but positively associated with patient prognosis [39]. Additionally, Yan et al. demonstrated that FGL1 can impede HCC progression through anchorage-dependent or -independent growth in vitro, while its knockdown enhances tumor proliferation [41]. Cao et al. identified the FGL1 receptor in HepG2 cells and revealed its role in regulating key cell cycle proteins, leading to cell cycle arrest and tumor growth inhibition. Specifically, FGL1 enhances the expression of p53 and p27 via intracellular pathways, leading to a decrease in CyclinD1 (CCND1) levels. This ultimately causes cell cycle arrest at the G0/G1 phase, effectively suppressing the proliferation of HCC [18]. In a diethylnitrosamine-induced liver cancer model, Hashemi et al. discovered that FGL1 knockout increased HCC incidence by activating protein kinase B (Akt) and mammalian target of rapamycin (mTOR) pathways. The knockout of FGL1 enhances Akt phosphorylation, leading to mTOR signaling pathway activation and subsequent phosphorylation of downstream proteins such as eIF4E-binding protein (4EBP1) and p70 ribosomal S6 kinase (p70S6K), ultimately promoting HCC proliferation. Interestingly, FGL1 knockout also increases the expression of tumor suppressor factors tripartite motif-containing protein 35 (TRIM35) and tumor necrosis factor superfamily 10b (TNFRSF10b), highlighting the diverse roles of FGL1 in HCC regulation [40]. Recent research has highlighted FGL1 as a ligand for the immune checkpoint LAG-3, showing that its interaction with LAG-3 on T cells induces T cell depletion, facilitating tumor immune evasion. Blocking FGL1 has been shown to enhance T cell anti-tumor activity [19]. Guo et al. conducted multiplex immunofluorescence analysis on HCC tissue samples, revealing higher FGL1 levels in tumors compared to adjacent tissues, positively correlating with LAG-3 expression and negatively correlating with CD8^+^ T cell abundance [115]. In advanced stages of HCC, FGL1 is inversely related to CD103 expression and is linked to a poor prognosis for HCC. The interaction between FGL1 and its receptor LAG-3 can result in the reduction of CD8^+^ tissue-resident memory T cells within tumors, facilitating immune evasion by the tumor [43]. Furthermore, research suggests that elevated levels of FGL1 in circulating tumor cells may be associated with resistance to programmed cell death protein-1 and its ligand PD-L1 (PD-1/PD-L1) immune checkpoint inhibitors [116].

It is crucial to explore the relationship between circulating FGL1 levels and HCC. Studies indicate that serum FGL1 levels are notably higher in patients with HBV-associated HCC compared to those with HBV-associated hepatitis and cirrhosis, particularly in cases where AFP is negative and the onset is early [117]. Analysis of circulating transcriptome samples from HCC patients also demonstrates a notable increase in FGL1 mRNA [118]. In addition, Yan et al. observed a link between FGL1 levels in circulating tumor cells and HCC tissues, indicating that patients with positive FGL1 expression tend to have higher TNM stages, distant metastases, and reduced postoperative survival [116]. These findings suggest that monitoring circulating FGL1 levels could potentially be a valuable biomarker for early detection of HCC.

HNF is a class of transcription factors that are highly expressed in hepatocytes and play a crucial role in maintaining hepatocyte morphology and function [119]. The down-regulation of HNF expression is often associated with the development of HCC, while overexpression of HNF-1α or HNF-4α can inhibit HCC occurrence and development, promoting tumor differentiation [120, 121]. Yu et al. identified HNF-1α as a key transcription factor in the upstream regulation of FGL1, which activates transcription and regulates HCC by recruiting HMGB1 and CREB to the FGL1 promoter [94]. Additionally, studies have shown that IL-6 can increase FGL1 expression in HepG2 cells in a dose-dependent manner [122]. Both HNF and IL-6 pathways are critical in hepatocyte regeneration and HCC. In a Colorectal Liver Metastases (CRLM) model, Li et al. demonstrated that tumor-associated macrophages (TAMs) activate the NF-κB signaling pathway in tumor cells by secreting TNF-α / IL-1β. Upregulation of OTU deubiquitinase 1 (OTUD1) stabilizes FGL1 through deubiquitination, further influencing the immune escape of metastatic tumors via the FGL1-LAG-3 axis [123] (Fig. 5B).

### The role of FGL2 in liver diseases

#### FGL2 in viral hepatitis

Viral hepatitis, an infectious disease caused by various hepatitis viruses, is characterized by hepatocyte necrosis and fibrin deposition [124]. In severe cases, it can progress to fulminant hepatitis (FH) or liver failure, and over time, chronic hepatitis may develop into cirrhosis and HCC [125]. Approximately 1.4 million people die annually due to viral hepatitis and its complications [126]. Research indicates that the expression level of FGL2 is closely linked to hepatitis severity, suggesting that FGL2 expression in PBMCs could serve as a potential marker for assessing hepatitis severity [127].

##### Acute viral hepatitis (AVH)

AVH is characterized by acute inflammation of the liver due to viral infection, resulting in massive hepatocyte necrosis and fibrin deposition that can progress rapidly to liver failure [128, 129]. Yang et al. identified that FGL2 expression in cells such as epithelial cells, macrophages, DCs, ECs, neutrophils, and a small amount in CD3^+^ T cells is involved in coagulation and complement activation during acute hepatitis caused by murine hepatitis virus strain 3 (MHV-3) infection [68, 130, 131]. The upregulation of mFGL2 in viral hepatitis promotes fibrin deposition, leading to blockage of blood flow in hepatic sinuses, hepatocyte necrosis, and worsening liver injury. Knockout of FGL2 in mice significantly reduces fibrin deposition, hepatocyte necrosis, and increases survival rates [132]. Additionally, Ding et al. reported that FGL2 plays a role in regulating liver injury in MHV-3-induced fulminant liver failure by selectively transcribing and expressing functional proteins in liver reticuloendothelial cells [133]. The upregulation of FGL2 may act as a protective mechanism against viral infection, regulating coagulation and immune response to maintain homeostasis, but excessive activation can also result in liver damage.

The activation of immune cells and excessive expression of cytokines are key factors in FH [134]. FGL2 plays a crucial role in AVH by modulating immune cell activity. Initial findings by Parr et al. revealed that MHV-3 infection enhances the secretion of FGL2 by macrophages [135]. Deleting FGL2 inhibits virus-induced FH by reducing inflammatory macrophage activation, impacting phagocytosis, and altering antigen presentation [81]. Infected macrophages release reactive oxygen species (ROS) and activate the NOD-like receptor pyrin domain containing 3 (NLRP3) inflammasome, leading to IL-1β secretion. This, in conjunction with TNF-α, activates the NF-κB pathway, upregulating FGL2 expression. Knocking out macrophage IL-1 receptor 1 (IL-1R1) significantly decreases FGL2 production, mitigating hepatocyte necrosis and fibrin deposition [136]. The immune system triggers apoptosis in virus-infected macrophages as a defense mechanism. However, FGL2 may impede this protective response. Research by Belyavskyi et al. demonstrated a negative correlation between MHV-3-induced macrophage apoptosis and FGL2 expression, as well as viral syncytial formation [137]. Knocking out B and T lymphocyte attenuator (BTLA) enhances macrophage apoptosis, reduces TNF-α production, decreases FGL2 expression, thereby alleviating liver inflammation and reducing mortality [130]. Ning et al. identified that nucleocapsid protein induces FGL2 expression through HNF-4α in MHV-3-infected macrophages [138]. MHV-3 infection can upregulate complement component 5a (C5a) in liver macrophages, leading to increased expression of FGL2 by binding with C5a receptor (C5aR) and activating ERK1/2 and p38 MAPK pathways [139, 140]. TNF-α and TNF receptor 1 (TNFR1) expression induced by MHV-3 infection further enhances FGL2 expression in hepatocytes, causing necrosis and apoptosis [141]. Interferon-gamma (IFN-γ) and TNF-α injection through the portal vein activates the STAT1 pathway, upregulating FGL2 expression and inducing hepatocyte apoptosis. Transcriptional regulation of FGL2 involves Sp1/Sp3 and STAT1/ purine-rich box-1 (PU.1) complexes [142]. PD-1 deficiency exacerbates MHV-3-induced liver damage by increasing FGL2 expression mediated by IFN-γ and TNF-α [143]. FGL2 overexpression activates NF-κB signaling pathway, promoting macrophage polarization towards pro-inflammatory phenotype and causing liver inflammatory injury [81].

**Fig. 6:**
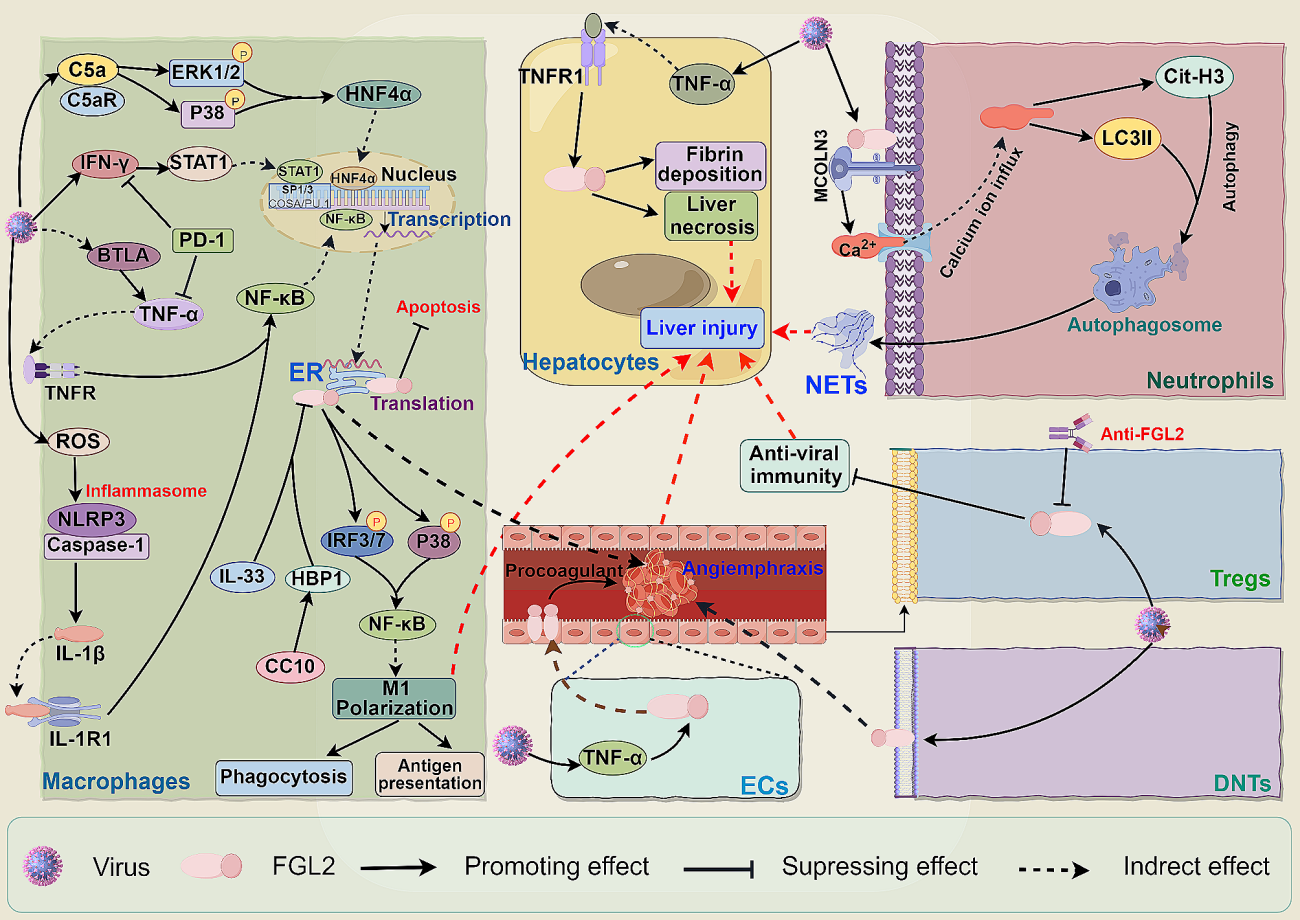
The roles of FGL2 in AVH. Following viral infection, TNF-α expression is upregulated in hepatocytes, leading to increased FGL2 expression in a TNF-R1-dependent manner. Additionally, in endothelial cells, heightened TNF-α levels further enhance FGL2 expression, induce procoagulant activity, and impede blood flow within hepatic sinusoids. Macrophages respond to viral activation by triggering STAT1 and HNF-4α through inflammatory factors and complement pathways. Furthermore, the virus activates the ROS/NLRP3/IL-1β pathway to boost FGL2 expression, which in turn promotes M1 polarization of macrophages via NF-κB pathway activation. Neutrophils experience FGL2/MCOLN3/Autophagy pathway activation by the virus, leading to the formation of neutrophil extracellular traps. Ultimately, this cascade culminates in hepatocyte necrosis and fibrin deposition, exacerbating acute hepatitis. Moreover, the virus induces FGL2 expression in Tregs, contributing to disease progression by suppressing antiviral immunity

FGL2 is identified as a regulator of Tregs, exerting its inhibitory effects on T cell function in a FOXP3-dependent manner [63]. Recent research has highlighted the immunosuppressive nature of the TIGIT^+^ Tregs subset, which selectively suppresses Th1 and Th17 cells by expressing sFGL2, thereby enhancing immunosuppression [144, 145]. In the context of MHV-3-induced fulminant viral hepatitis (FVH), FGL2 has been shown to increase host susceptibility. Targeting FGL2 with an anti-FGL2 antibody can attenuate Treg activity, consequently mitigating liver injury [146]. Notably, during MHV-3-induced FVH, there is a significant rise in DNTs, with splenic DNTs expressing high levels of CD69 being recruited to the liver by infected hepatocytes, where they upregulate mFGL2 expression, contributing to liver cell necrosis and fibrin deposition [70]. Furthermore, the activation of the FGL2- mucolipin 3 (MCOLN3)-Autophagy axis leads to the release of Neutrophil extracellular traps (NETs) through the upregulation of mFGL2, exacerbating inflammatory damage and fibrin deposition. Additionally, FGL2 can induce the expression of C-X-C motif chemokine ligand (CXCL) 1, CXCL2, C-X-C chemokine receptor (CXCR) 2, and intercellular adhesion molecule-1 (ICAM-1), further facilitating neutrophil recruitment to the liver and increasing NETs formation, thus perpetuating a detrimental cycle [68]. These insights underscore the pivotal role of FGL2 in AVH by modulating the functions of macrophages, Tregs, neutrophils, and distinct T cell subtypes (Fig. 6).

##### Chronic viral hepatitis (CVH) and cirrhosis

**Fig. 7:**
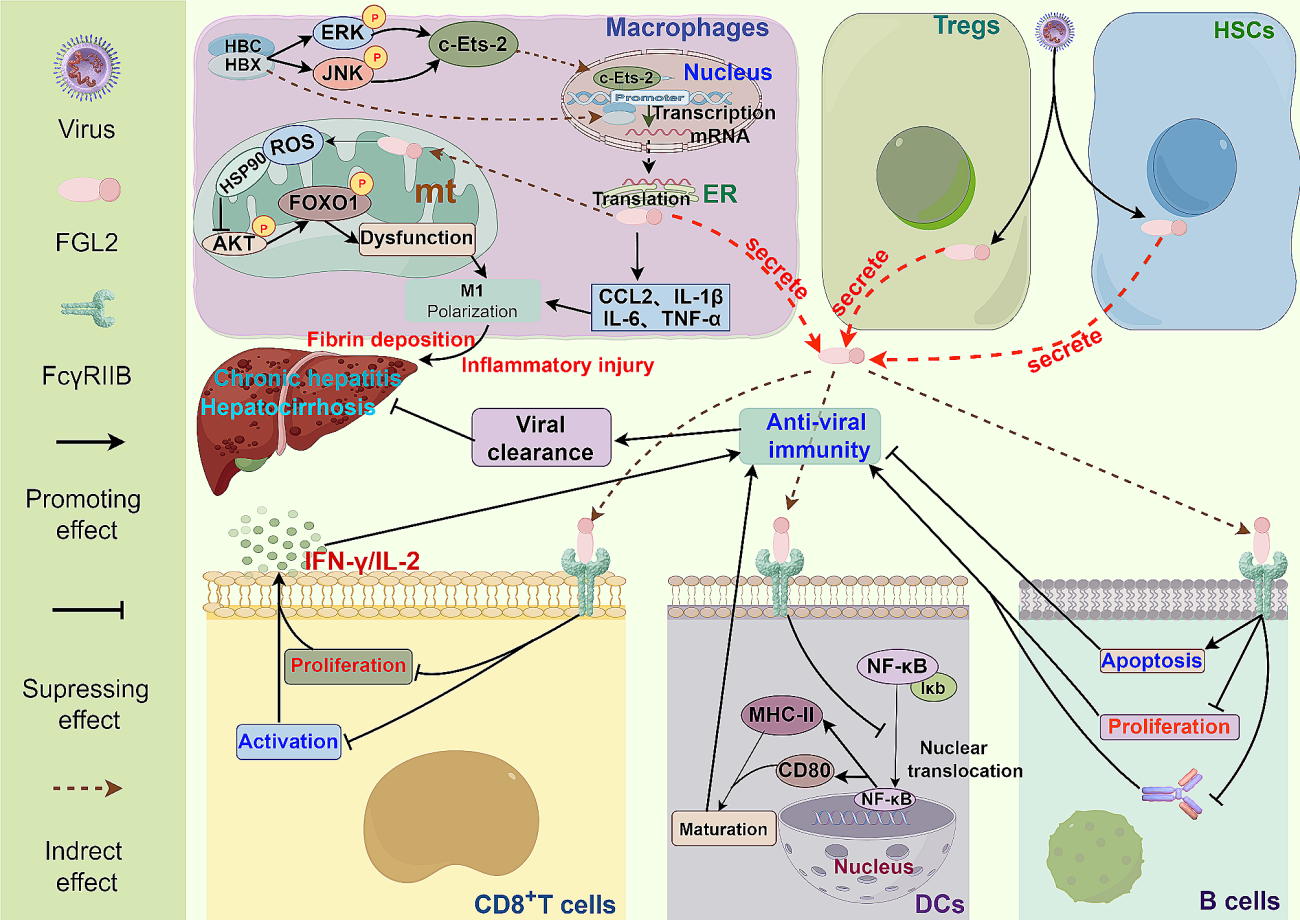
The roles of FGL2 in CVH and Cirrhosis. In the context of viral infection, viral proteins in CVH can interact with promoters or activate ERK or JNK pathways to facilitate c-Ets-2 nuclear translocation, thereby upregulating FGL2 expression in macrophages. This increased FGL2 expression can trigger the production of inflammatory factors, induce mitochondrial dysfunction, and ultimately drive macrophage M1 polarization. This polarization contributes to hepatocyte inflammatory injury and fibrin deposition. Additionally, viral infection can elevate FGL2 levels in Tregs and HSCs, which in turn suppress antiviral immunity and promote disease progression and chronicity by inhibiting the function of DCs, B cells, and CD8^+^ T cells

CVH is a long-term liver inflammation caused by hepatitis viruses, primarily HBV and HCV infections [147]. FGL2, a multifunctional protein, plays a role in promoting liver fibrin deposition and accelerating liver fibrosis, while also suppressing the immune system’s ability to clear the virus, leading to disease progression [28] (Fig. 7). Research indicates that FGL2 is highly expressed in macrophages, ECs, and Tregs of CVH patients [72]. Furthermore, FGL2 expression is notably increased in PBMCs of individuals with chronic hepatitis B (CHB) [148]. FGL2 influences hepatocyte susceptibility to viruses by modulating various immune cells [149]. Elevated levels of plasma sFGL2 in HBV-infected individuals are positively associated with HBV-DNA load, aspartate transaminase (AST) levels, disease progression, and clinical outcomes [150]. In chronic hepatitis C (CHC), sFGL2 levels correlate with viral titer and liver inflammation, decreasing after effective antiviral therapy. Patients with HCV-associated cirrhosis exhibit higher sFGL2 levels compared to those without cirrhosis or with non-active end-stage alcoholic cirrhosis [149]. Previous research has demonstrated a correlation between plasma FGL2 levels and HCV virus replication, indicating that individuals with elevated FGL2 levels may have a more active form of HCV and are less responsive to treatment [55]. These findings suggest that plasma sFGL2 levels could serve as a valuable biomarker for assessing the severity of viral hepatitis and evaluating treatment outcomes [30, 149]. Furthermore, studies by Yu et al. and Sun et al. have shown that FGL2 expression is heightened in patients with cirrhosis, particularly during the recovery phase of severe hepatitis B, implying a potential role of FGL2 in the fibrotic process [31, 78]. Additionally, the expression of FGL2 has been linked to the degree of inflammation and fibrosis in the livers of HBV-infected patients and experimental models, with the deletion of the FGL2 gene showing promise in reducing liver inflammation and fibrosis progression [151].

HBV infection induces the secretion of sFGL2 in Tregs, leading to a reduction in IFN-γ and IL-2 production by suppressing the proliferation of CD8^+^ T cells, thereby impairing the immune system’s ability to clear the virus [152]. Similarly, following HCV infection, CD4^+^CD25^+^Foxp3^+^ Tregs inhibit the immune response to viral infection by releasing sFGL2 [149]. However, some research suggests that sFGL2 can trigger hepatocytes apoptosis, contributing to the development of chronic hepatitis [153]. Han et al. demonstrated that HBV can directly interact with the promoter of the FGL2 gene in THP-1 cells. Furthermore, HBV proteins HBc and HBx activate ERK and JNK pathways, facilitating c-Ets-2 nuclear translocation and enhancing FGL2 transcription [154]. Overexpression of FGL2 in macrophage mitochondria increases reactive oxygen species (ROS) production, which binds to heat shock protein 90 (HSP90) and inhibits Akt phosphorylation and downstream forkhead box O1 (FoxO1), leading to mitochondrial dysfunction. Additionally, FGL2 upregulates the expression of C-C motif chemokine ligand 2 (CCL2), IL-1β, IL-6, and TNF-α, promoting the differentiation of macrophages into M1-like pro-inflammatory macrophages and exacerbating liver inflammation and fibrosis [151]. Studies have also shown that sFGL2, serves as a novel effector molecule of activated hepatic stellate cells (HSCs), can inhibit CD8^+^ T cell proliferation and IFN-γ generation, dampening the antiviral immune response and driving liver fibrosis progression [31].

#### FGL2 in NAFLD

Plasma sFGL2 levels were found to be elevated in patients with NAFLD compared to healthy controls [155]. Hu et al. also observed a significant increase in FGL2 expression in the livers of patients with NASH and mouse models. The expression of FGL2 progressively rose with the advancement of NASH. In vitro experiments showed that primary hepatocytes cultured with FGL2-knockout bone marrow-derived macrophages (BMDMs) conditioned medium exhibited lower fat deposition compared to those cultured with wild-type BMDMs. Free fatty acids (FFAs) were found to enhance FGL2 expression in the liver, leading to its interaction with TLR4 on macrophages’ surface, subsequently activating the NF-κB and p38-MAPK pathways through the adapter protein myeloid differentiation factor-88 (MyD88). Furthermore, macrophages were shown to release ROS and activate the NLRP3 inflammasome, ultimately inducing the production of pro-inflammatory cytokines (TNF-α, IL-6, CCL2, IL-1β, IL-18), thereby exacerbating liver inflammation and lipid metabolism disturbances in NASH [82] (Fig. 4).

#### FGL2 in HCC

The ability of tumors to evade immune surveillance and control is a crucial characteristic. Tregs, as key immune cells in the TME, regulate the function of effector T cells and play a significant role in tumor development [156, 157]. FGL2, an essential effector of Tregs, inhibits T cell proliferation and cytotoxic effects by down-regulating antigen-presenting function through binding to FcγRIIB on APCs [158, 159]. Targeting the FGL2-FcγRIIB pathway could potentially be a groundbreaking approach in tumor immunotherapy [160].

**Fig. 8:**
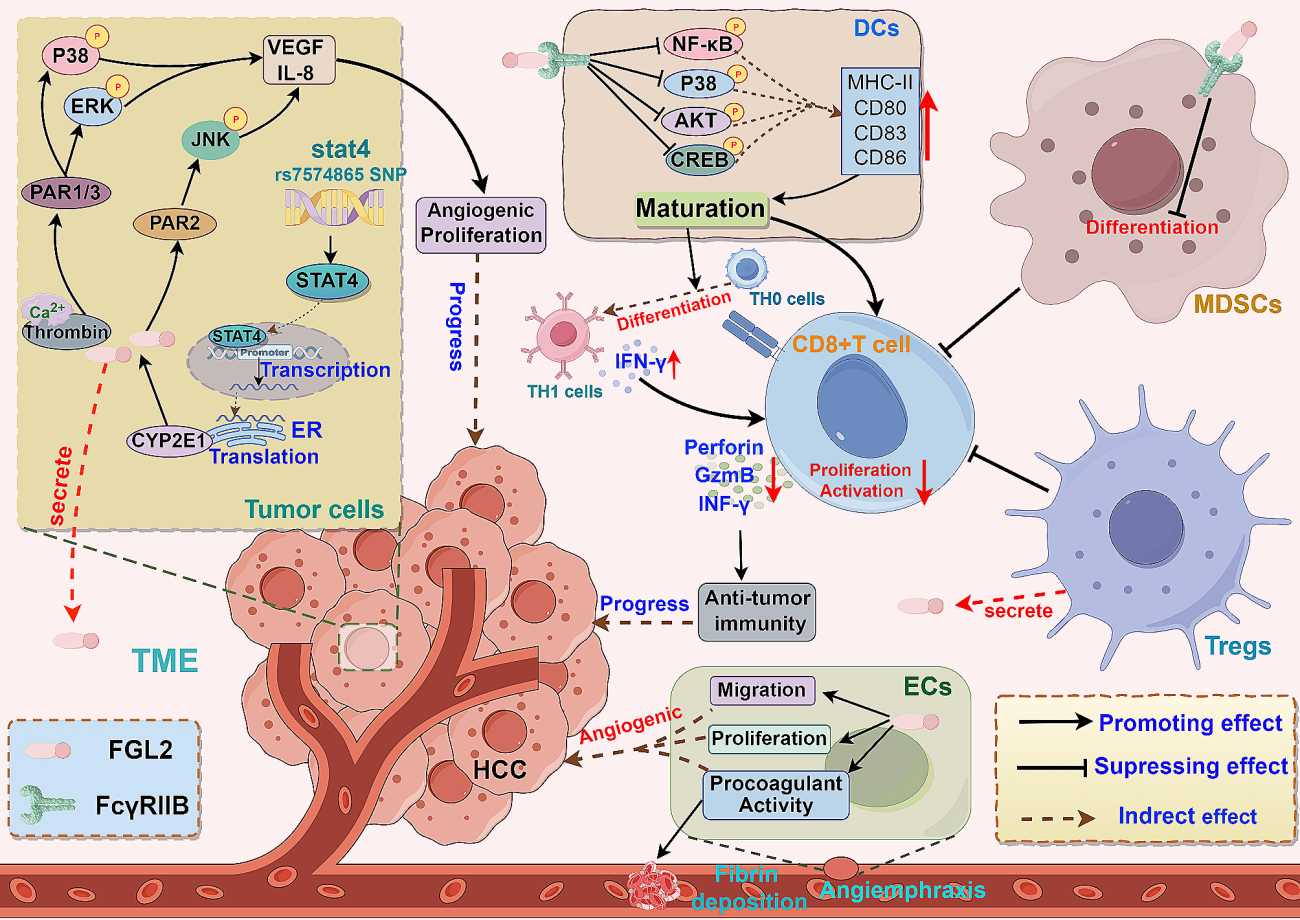
The roles of FGL2 in HCC. The STAT4 gene polymorphism in tumor cells promotes the expression of FGL2, which aids in tumor progression by enhancing tumor cell proliferation and angiogenesis. FGL2 binds to FcγRIIB on dendritic cells’ surface, leading to the inhibition of dendritic cell maturation and the differentiation of TH0 cells to TH1 cells, consequently hindering the activation of CD8^+^ T cells. Moreover, FGL2 also obstructs MDSCs differentiation, thereby contributing to immunosuppression by impeding CD8^+^ T cells. Furthermore, heightened FGL2 expression in endothelial cells stimulates endothelial cell proliferation and migration, amplifies procoagulant activity

Multiple studies have demonstrated that FGL2 is highly expressed in liver cancer tissues and plays a crucial role in tumor progression by activating specific signaling pathways and modulating immune cell functions [31, 80] (Fig. 8). Additionally, the common Single Nucleotide Polymorphism (SNP) stat4 rs7574865 has been significantly associated with the development of HCC [161]. Wang et al. identified that homozygous polymorphisms of stat4 rs7574865 mutants result in elevated STAT4 expression, leading to increased Cytochrome P450 2E1 (CYP2E1) expression by regulating its promoter region. This, in turn, up-regulates FGL2 expression, contributing to HCC progression. Furthermore, STAT4 silencing induces apoptosis in HepG2 cells, impeding their invasion and migration [162]. The secretion of sFGL2 by LX2 cells suppresses CD8^+^ T cell proliferation and IFN-γ production in a dose-dependent manner, consequently impeding anti-tumor immunity in HCC patients [31]. Yang et al. observed that sFGL2 reduces the phosphorylation of Akt, NF-κB, CREB, and p38 MAPK, down-regulates the expression of MHCII, CD40, CD80, CD86, and CD83 on BMDCs, inhibits DC activation, and promotes Treg proliferation. Moreover, sFGL2 inhibits T cell proliferation, TH0 cell differentiation into TH1 cells, decreases perforin and granzyme B production, and ultimately facilitates tumor progression. Blocking sFGL2 in orthotopic and subcutaneous transplanted HCC models enhances DC maturation, augments the number and function of CD8^+^ T cells in the tumor infiltrating area, and suppresses tumor growth [145]. A study on gliomas revealed that targeting FGL2 depletion activates anti-tumor activity of CD8^+^T cells by facilitating the differentiation of CD103^+^ DCs [158]. FGL2 has been shown to enhance the accumulation of myeloid suppressor cells (MDSCs) in the TME, thus promoting the progression of HCC [163]. Additional research has demonstrated that FGL2 contributes to tumor advancement in HCC by modulating the tumor cell cycle and angiogenesis. Knocking down FGL2 in the HCCLM6 cell line can suppress the activation of PAR1/3, leading to inhibition of downstream ERK and P38 MAPK phosphorylation. Furthermore, FGL2 knockdown hinders the activation of PAR2 and weakens downstream JNK phosphorylation, resulting in tumor cell cycle arrest, reduced expression of vascular endothelial growth factor (VEGF) and IL-8, and inhibition of tumor proliferation and angiogenesis [80]. Recent findings from our team suggest that FGL2 promotes ROS production and activates the X-box binding protein 1 (XBP1) pathway by regulating cholesterol metabolism, thereby fostering MDSCs accumulation and immunosuppression. Depletion of FGL2 diminishes the accumulation of granulocytic MDSCs and boosts the anti-tumor response of CD8^+^ T cells [164].

FGL2 exhibits prothrombin activity, can initiate the clotting cascade, and potentially enhances tumor progression. Within tumor tissues, FGL2 can enhance fibrin deposition, act as a scaffold for angiogenesis, and stimulate ECs proliferation and migration [165]. Su et al. demonstrated that IFN-γ and IL-2 can upregulate FGL2 expression in macrophages, contributing to a hypercoagulable state in HCC and facilitating tumor angiogenesis and metastasis [65]. This similar mechanism has also been observed in glioma, where FGL2 interacts with thrombin and tissue factors to induce a hypercoagulable state within the tumor, ultimately promoting tumor angiogenesis and metastasis [166].

#### FGL2 in other liver diseases

Recent research has shown that FGL2 plays a role in various liver-related conditions such as Autoimmune hepatitis (AIH), DILI, alcoholic hepatitis, liver parasitic infection, and liver ischemia-reperfusion injury. In autoimmune diseases, Tregs are recruited to the inflamed site and contribute to the expression of FGL2 [160]. Some studies suggest that Tregs inhibit the function of CD8^+^ T cells and Tc17 cells through sFGL2, potentially limiting the progression of AIH [167]. Moreover, sFGL2 released by MSCs can mitigate immune damage in AIH by promoting the conversion of CD4^+^ T cells into Tregs [168]. Conversely, in CCL4-induced DILI, increased FGL2 expression worsens liver injury [169]. In cases of alcoholic hepatitis, FGL2 levels rise in liver macrophages, interacting with PKM2 to enhance glycolysis in proinflammatory macrophages and exacerbate liver damage [170]. Additionally, during malaria sporophytic infection, mFGL2 expression increases in liver Kupffer cells and ECs, leading to elevated inflammatory cytokine levels [171]. FGL2-induced apoptosis of sinusoidal endothelial cells and hepatocytes, through binding to the FcγRIIB receptor, contributes to liver ischemia-reperfusion injury [153]. Despite these findings, further research is required to fully elucidate the precise mechanisms of FGL2 in these disease contexts.

#### FGL2 in liver transplantation

Liver transplantation is a well-established treatment for end-stage liver disease, however, rejection remains a major challenge for both the allografts and recipients in the long term [172]. The primary cause of acute rejection (AR) is the persistent activation of immune cells, resulting in tissue cell damage. T cells are key players in the specific immune response during acute allograft rejection [91, 173], while Tregs are essential for inducing and maintaining graft tolerance [174, 175]. Following organ transplantation, the expression level of sFGL2, an effector of Tregs, significantly rises, contributing to transplantation tolerance through immune regulation and inflammatory response [91]. Research by Pan et al. demonstrated that up-regulation of sFGL2 in rat liver transplantation models led to M2 polarization of Kupffer cells, ameliorating AR, and extending survival by enhancing the secretion of anti-inflammatory cytokines IL-10 and TGF-β [176, 177]. Moreover, in liver allograft models that were tolerated, sFGL2 levels increased notably during cell rejection and post-rejection stages. A biomarker panel incorporating Tregs-related genes, such as FGL2, was developed to differentiate between tolerated and rejected allografts. Monitoring the local expression of FGL2 dynamically in allografts could be crucial for identifying transplantation tolerance [178].

## Targeting fibrinogen-like proteins for liver disease treatment

Current consensus suggests that fibrinogen-like proteins are believed to play a significant role in the initiation and progression of various liver diseases. While research in this area is still in its initial phases, there is considerable optimism regarding the potential of Fibrinogen-like proteins as novel therapeutic targets for liver disease.

### FGL1

In acute liver injury models induced by D-Gal or CCL4, the administration of recombinant FGL1 has been shown to decrease the severity of liver injury [179]. Moreover, in the fulminant hepatic failure (FHF) model induced by D-Gal and CCL4, recombinant FGL1 also notably decreased hepatocyte apoptosis and stimulated hepatocyte regeneration. Knockdown of FGL1 in vivo has been found to exacerbate D-Gal-induced liver injury [99]. Additionally, in NAFLD, the targeted deletion of FGL1 led to a reduction in HDF-induced hepatic lipid accumulation, improvement in hepatic steatosis, and a decrease in NAS [52].

Targeting FGL1 in HCC has shown promising therapeutic potential. Research indicates that Norcantharidin (NCTD) and Oxysophocarpine can downregulate FGL1 expression by inhibiting the IL-6/JAK2/STAT3 pathway, preventing FGL1 from binding to LAG-3, and enhancing the anti-tumor activity of CD8^+^ T cells. Moreover, NCTD has been observed to enhance sorafenib-induced tumor immune reactivation, thereby boosting its anti-tumor efficacy [42, 44]. Post-translational modifications of FGL1 are crucial in HCC regulation. Studies have revealed that 2-Cyano-3-[5-(2,5-dichlorophenyl)-2-furanyl]-N-5-quinolinyl-2-propenamide (AGK2) (an inhibitor of the deacetylase SIRT2) and aspirin can induce FGL1^K98^ acetylation, leading to its degradation and improving the effectiveness of HCC immunotherapy [47]. Notably, FGL1 has been implicated in sorafenib resistance through ERK and autophagy pathways, with high FGL1 expression associated with reduced sorafenib sensitivity [180]. Furthermore, FGL1 has been linked to gefitinib resistance in non-small cell lung cancer by modulating the poly (ADP-ribose) polymerase 1 (PARP1)/caspase-3 pathway [181]. In CRLM, inhibiting FGL1 secretion with Benzethonium chloride has been shown to inhibit tumor growth [123]. Overall, the role of FGL1 in tumor-targeted therapy is intricate and multifaceted.

### FGL2

Given the critical regulatory role of FGL2 in viral hepatitis and HCC, targeting FGL2 may offer new therapeutic avenues. Khattar et al. discovered that while deleting FGL2 initially increased viral replication in LCMV-induced AVH, it also enhanced the response of anti-viral adaptive T & B cells, thereby boosting anti-viral effectiveness [182]. Similarly, in MHV-3-induced FVH, treatment with anti-FGL2 antibodies or antisense plasmids targeting FGL2 gene exon I could lessen severity and mortality [183]. Introducing a recombinant adenovirus carrying microRNA targeting FGL2 in vivo reduced liver inflammation and improved mouse survival rates [70]. Combined intervention using adenovirus-mediated microRNA targeting mFgl2, mFas, and mTNFR1 significantly ameliorated liver inflammatory infiltration, reduced hepatocyte necrosis and apoptosis, and promoted hepatitis recovery [184]. Similar results were achieved with the dual-interfering shRNA plasmid targeting mFgl2 and mTNFR1 in MHV-3-induced FH in mice [185]. Furthermore, studies have shown that Clara cell 10-kDa protein (CC10) and IL-33 can mitigate liver injury in FH by suppressing FGL2 expression [186, 187]. In CVH, the use of anti-FGL2 antibodies alleviates its inhibitory effect on T cells, enhances the antiviral immune response, and reduces liver fibrosis [31, 152].

In a study by Wang et al., adenovirus-mediated miRNA-targeted silencing of the FGL2 gene was used to inhibit HCC growth and angiogenesis in a mouse model [188]. Similarly, in subcutaneous transplanted HCC models, inhibiting tumor growth was achieved through the use of the anti-FGL2 antibody or FGL2 knockout [145]. Our research team discovered that combining anti-FGL2 and anti-PD-L1 could enhance the effectiveness of colon cancer immunotherapy [164], supporting FGL2 as a promising target for combined immunotherapy. Furthermore, targeting FGL2 has shown efficacy in glioma, lung cancer, and melanoma [60]. Studies have also demonstrated that deleting FGL2 can reduce liver steatosis and inflammation in diet-induced NASH models [82]. In cases of alcoholic hepatitis, depleting FGL2 has been found to improve ethanol-induced hepatic steatosis, oxidative injury, and proinflammatory cytokines [170]. Targeting FGL2 in liver macrophages may hold promise as an intervention for NAFLD and alcoholic hepatitis.

## Conclusions and perspectives

As a pivotal hepatokine, FGL1 plays a crucial role in liver damage repair. Following various physical or chemical insults to the liver, the expression of FGL1 is notably upregulated, aiding in the reduction of hepatocyte apoptosis and promotion of hepatocyte regeneration for tissue repair. Moreover, Demchev et al. discovered that signals triggered post subtotal hepatectomy also boost FGL1 expression in brown adipose tissue, indicating FGL1’s role as a communication link between injured liver and adipose tissue [15]. While FGL1 can mitigate liver injury caused by CCL4 and D-Gal and dampen the inflammatory response, it may also lead to lipid accumulation in the liver, contributing to the onset and progression of NAFLD. FGL1 exhibits a dual regulatory function in HCC, inhibiting tumor cell proliferation and influencing sorafenib resistance while simultaneously interacting with LAG-3 to foster an immunosuppressive TME, thereby supporting tumor advancement. This dual nature complicates the targeting of FGL1 as a therapeutic approach for HCC. Future investigations should delve deeper into the regulatory mechanisms of FGL1. Only through the development of drugs that selectively target the distinct regulatory pathways of FGL1 can the clinical utilization of FGL1 be feasible. Furthermore, FGL1 has been implicated in the regulation of epithelial-mesenchymal transition (EMT) and angiogenesis in various cancers such as lung cancer [189], laryngeal cancer [190], and renal clear cell carcinoma [191]. In a study focusing on kidney cancer, the FGL1/ carbonic anhydrase IX (CAIX) dual-target vaccine exhibited anti-tumor effects by enhancing the immune activity of CD8^+^ T cells [192]. This has led us to consider whether FGL1 also regulates tumor progression through other mechanisms and whether combination therapies based on FGL1 should also be attempted in HCC. Additionally, FGL1 has demonstrated promise as a biomarker for evaluating the severity and prognosis of liver injury caused by radiation, drugs, metabolic disorders, and HCC. These findings have expanded the horizons of FGL1 research in the field of liver disease.

Numerous studies over the past few decades have highlighted the significant regulatory role of FGL2 in various liver diseases. Initially recognized for its procoagulant action during viral infections, FGL2 has more recently been found to play a crucial role in immune regulation as well. In AVH, FGL2 induces liver cell necrosis and fibrin deposition by impacting coagulation and immune responses. In CVH, FGL2 plays a key role in suppressing the antiviral immune response through modulation of immune cell functions, impairing the body’s ability to eliminate the virus. This can result in a persistent disease course, ultimately leading to cirrhosis and liver cancer. Monitoring FGL2 levels is essential for evaluating the severity of viral hepatitis. In HCC, the intricate and diverse regulatory mechanisms of FGL2 are evident, as it not only creates an immunosuppressive microenvironment by influencing various immune cells, but also facilitates tumor angiogenesis and metastasis through its prothrombin activity. The multifaceted nature of FGL2 is also observed in NAFLD and liver transplantation. In the context of NAFLD, FGL2 has been found to regulate macrophages, leading to the promotion of liver inflammation. Conversely, in liver transplantation, FGL2 exhibits an immunosuppressive function, protecting both the host and the graft. Monitoring the levels of FGL2 dynamically proves to be advantageous in evaluating transplant tolerance.

The potential of FGL1 and FGL2 as therapeutic targets for liver disease is promising, given their versatility for targeted therapies. However, understanding their complex roles in the liver and elucidating their mechanisms of action in different liver diseases remains a challenge. Current research is still in its early stages, with uncertainties about clinical translation. Future studies should focus on comprehensively exploring their interactions with hepatocytes and immune cells, identifying the signaling pathways involved, and understanding their regulatory networks in various liver diseases. Additionally, investigating combination therapies involving FGL1 or FGL2 in liver cancer and other liver diseases is crucial for enhancing treatment efficacy. Investigating the regulatory mechanisms of FGL1 and FGL2 in physiological and pathological conditions on a global scale can offer valuable insights for the treatment of liver diseases. Moreover, studying the potential of fibrinogen-like proteins as biomarkers for liver diseases holds immense value for diagnosing and assessing the prognosis of these conditions. In conclusion, further investigation into novel therapeutic strategies targeting FGL1 and FGL2 in liver diseases is warranted.

## Data Availability

No datasets were generated or analysed during the current study.

## Abbreviations

ACC-1: Acetyl-CoA carboxylase-1
ADAM: A disintegrin and metalloproteinase
AGK2: 2-Cyano-3-[5-(2,5-dichlorophenyl)-2-furanyl]-N-5-quinolinyl-2-propenamide
AIH: Autoimmune hepatitis
AP1: Activator protein-1
APCs: Antigen-presenting cells
AR: Acute rejection
AST: Aspartate transaminase
AVH: Acute Viral hepatitis
BMDMs: Bone marrow-derived macrophages
BMSCs: Bone marrow mesenchymal stem cells
CAIX: Carbonic anhydrase IX
CC10: Clara cell 10-kDa protein
CCD: Coiled-coil domain
CCL2: C-C motif chemokine ligand 2
CCL4: Carbon tetrachloride
CCND1: CyclinD1
CHB: Chronic hepatitis B
CHC: Chronic hepatitis C
Cit-H3: Citruillinated histone H3
CMECs: Cerebral Microvascular Endothelial Cells
CRLM: Colorectal Liver Metastases
CREB: cAMP response element-binding protein
CTLs: Cytotoxic T lymphocytes
C5a: Complement component 5a
C5aR: C5a receptor
CVH: Chronic Viral hepatitis
CXCL: C-X-C motif chemokine ligand
CXCR: C-X-C chemokine receptor
CYP2E1: Cytochrome P450 2E1
C/EBP: CCAAT/enhancer binding protein
D-Gal: D-galactosamine
DC: Dendritic cell
DILI: Drug-induced liver injury
DNTs: CD3^+^CD4^−^CD8^−^T cells
ECs: Endothelial cells
EGFR: Epidermal growth factor receptor
ERK: Extracellular signal-regulated kinases
ETS: E26 transformation-specific
FAS: Fatty acid synthase
FcγR: Fc gamma receptor
FcγRIIB: Fc gamma receptor
FcγRIII: Fc gamma receptor III
FD: Fibrinogen-like structural domain
FGL1: Fibrinogen-like protein 1
FGL2: Fibrinogen-like protein 2
FH: Fulminant hepatitis
FHF: Fulminant hepatic failure
FoxO1: Forkhead box O1
FREPs: Fibrinogen-related proteins
FReDs: Fibrinogen-related domains
FVH: Fulminant viral hepatitis
HBP1: HMG-box transcription factor 1
HBV: Hepatitis B virus
HCC: Hepatocellular carcinoma
HCV: Hepatitis C virus
HFREP-1: Hepatocyte-derived fibrinogen-related protein 1
HFD: High-fat diet
HMGB1: High mobility group box 1
HNF: Hepatocyte nuclear factor
HPS: Hepassocin
HSCs: Hepatic stellate cells
HSP90: Heat shock protein 90
ICAM-1: Intercellular adhesion molecule-1
IELs: Intraepithelial lymphocytes
IFN-γ: Interferon-gamma
IL: Interleukin
IL-1R1: Interleukin-1 receptor 1
IL-6R: IL-6 Receptor
ITAM: Immunoreceptor tyrosine-based activation motif
ITIM: Immunoreceptor tyrosine-based inhibition motif
JAK2: Janus kinase 2
JNKs: C-Jun N-terminal kinases
LAG-3: Lymphocyte activation gene 3
LC3II: Microtubule-associated proteins light chain 3 II
LPS: Lipopolysaccharide
MAPK: Mitogen-activated protein kinase
MCOLN3: Mucolipin 3
MDSCs: Myeloid suppressor cells
MHVs: Murine hepatitis virus strains
mFGL2: Transmembrane FGL2
MSC: Mesenchymal stem cell
mSREBP-1: Mature sterol regulatory element-binding protein 1
mTOR: Mammalian target of rapamycin
MyD88: Myeloid differentiation factor-88
NAFL: Nonalcoholic fatty liver
NAFLD: Non-alcoholic fatty liver disease
NASH: Nonalcoholic steatohepatitis
NAS: NAFLD activity score
NCTD: Norcantharidin
NETs: Neutrophil extracellular traps
NF-κB: Nuclear factor-kappaB
OTUD1: Upregulation of OTU deubiquitinase 1
PARP1: Poly (ADP-ribose) polymerase 1
PARs: Protease-activated receptors
PBMCs: Peripheral blood mononuclear cells
PD-1: Programmed cell death protein-1
PD-L1: Programmed death-ligand 1
PHx: Partial hepatectomy
PP2A: Protein Phosphatase 2 A
PPARα: Peroxisome proliferator-activated receptor alpha
PRRs: Pattern Recognition Receptors
PU.1: Purine-rich box-1
p70S6K: p70 ribosomal S6 kinase
RILD: Radiation-induced liver damage
sFGL2: Soluble FGL2
SNP: Single Nucleotide Polymorphism
SP1: Specificity protein 1
SREBP-2: Sterol regulatory element-binding protein 2
STAT3: Signal transducer and activator of transcription 3
TAMs: Tumor-associated macrophages
TCF1: T cell factor 1
TME: Tumor microenvironment
TLR4: Toll-like receptor 4
TLRs: Toll-like Receptors
TNF-α: Tumor necrosis factor-alpha
TNFR1: TNF receptor 1
TNFRSF10b: Tumor necrosis factor superfamily 10b
TRAF6: Tumor necrosis factor receptor-associated factor 6
TRIM35: Tripartite motif-containing protein 35
UFAs: Unsaturated fatty acids
VEGF: Vascular endothelial growth factor
WHO: World Health Organization
XBP1: X-box binding protein 1
4EBP1: eIF4E-binding protein
